# Editorial: Social Impact of Research in Psychology

**DOI:** 10.3389/fpsyg.2021.663817

**Published:** 2021-09-27

**Authors:** Erica R. Halverson, Marta Soler-Gallart, Sara Cadavid

**Affiliations:** ^1^Department of Curriculum and Instruction, University of Wisconsin-Madison, Madison, WI, United States; ^2^Department of Sociology, University of Barcelona, Barcelona, Spain; ^3^School of Medicine and Health Sciences, Universidad del Rosario, Bogotá, Colombia

**Keywords:** social impact, educational psychology, research methodologies, effective interventions, psychological science

Currently, one key European priority is to make scientific evidence with social impact available to the public. The Directorate-General for Research and Innovation (European Commission) published the Expert Report “Monitoring the impact of EU Framework Programmes” (Besseelaar et al., 2018) clarifying the impact requirements for research proposals submitted to Horizon Europe. By prioritizing social impact and making scientific evidence available to society, Article 27 of the Universal Declaration of Human Rights, which states that everyone has the right to share in scientific advancement and its benefits, becomes effective.

Hence, this is an ideal moment to clarify the effective and potential social impact (Pulido et al., 2018; Aiello et al., 2020) that research in psychology has had until now and will have in the future. The aim of this Research Topic is to collect those studies that demonstrate, with evidence, how psychological research in diverse areas of the discipline can address crucial needs in society and solve most of the pressing social problems, thus achieving social impact. The articles composing this Research Topic represent a great variety of methodologies and fields within psychological research, such as education, gender, violence, or well-being. It also includes methodological contributions on how to gather the social impact (Gómez et al., 2019; Sordé Martí et al., 2020) of psychological research using different tools and data sources.

Some studies in this Research Topic have focused on education, showing that educational psychology is providing the citizenry with increasing evidence that contributes to achieving social impact worldwide. Educational psychology is placing great endeavors in advancing knowledge and evidence fostering all children's learning, academic success, prosocial behavior, well-being, and, ultimately, providing them with opportunities to have a flourishing life (García-Carrión et al., 2019). One of the areas of study in educational psychology is the impact of dialogue and communicative interactions with diverse others on learning and development. By reviewing different schools of thought on dialogic teaching and learning, García-Carrión et al. explored the social improvements of dialogic education with a focus on aspects such as academic success and social cohesion. The article discussed some of the hindrances to its further social impact, as well as the affordances of communicative mixed methods in this field to achieve social impact. Along the lines of dialogic education, Duque et al. explored the social impact of research focused on analyzing the benefits of successful educational actions for students with special needs. Through 10 case studies and in-depth interviews, their analysis showed the identification of the benefits of interactive learning environments in mainstream schools, the spread of these successful actions to more mainstream schools, and their transfer to special schools, improving educational opportunities for students with special needs. Research in educational psychology has also explored the impact of dialogue and interactions in informal learning contexts. An example from this Research Topic is Meng et al.'s study on mothers' and grandmothers' influence when co-viewing cartoons with children, where 89 Chinese parents and grandparents participated. Results showed that mothers think cartoons have a very high influence on children's health and put more restrictions on the content of the cartoons than grandmothers and that children's interactions with mothers are more based on experience proofs than with grandmothers. The current Research Topic includes a critical, longitudinal, and participatory ethnography on the narrative of a female American youth developing a culture of criticality. In this study, Calabrese Barton et al. showed how the space between person and institution worked as an incubator for the youth's engagement in a pathway toward becoming *somebody* in STEM.

In addition, educational psychology has served as the basis for educational interventions that are achieving social impact. Alongside the dialogic education approach explored in the aforementioned studies, Rodriguez et al. analyzed a successful dialogue-based teacher training program in which teachers read and critically discuss educational theory and evidence. Through a questionnaire aimed at 69 teachers in Spain, they observed the seminar's impact in promoting teachers' self-efficacy and argumentative skills and in transferring this new knowledge to their profession, transforming their everyday practice. Furthermore, the impact of research on dialogic teaching and learning interventions has been transferred to different fields, such as organizations and leadership. One of the studies presented in this Research Topic, conducted by Campos et al., analyzed the impact of an excellence EMBA program regarding its organizational components, particularly its goals and composition. Through an open-ended questionnaire addressed to 28 alumni and seven current students in the program, findings revealed that participants improved their leadership skills and aptitudes and placed great importance on dialogue and communication as key abilities among leaders. Along the line of educational programs based on evidence from psychology, Trigos-Carrillo et al. presented findings from a service-learning program in Colombia in which 27 psychology students and two faculty co-existed and shared daily life activities with a community of former guerrilla members. They resorted to Participatory Action Research to explore the program's impact as a potential avenue for bringing rural and urban communities closer and toward peacebuilding by developing students' cultural humility. Shedding light on educational psychology's contributions to gaming programs, Chau et al. showed the impact of the Wise IT-use program developed by them in order to tackle Internet gaming disorders and risky online behaviors. Validated questionnaires were administered to 248 primary students in Hong Kong before and after participating in the program. Results showed the program successfully mitigated the symptoms of Internet gaming disorder and increased emotional well-being among participants.

On the other hand, research in psychology has attained social impact in the field of violence-related issues, as other articles within this Research Topic evidence. Some have focused on sexual-affective and gender-based violence. Flecha et al. brought to light the contributions of psychology to the existing theoretical, scientific, and social debate on sexual consent, addressing the new challenges that have arisen and presenting a new research line that takes on some of these challenges, further propelling the impact of psychology on this issue. Placing the responsibility for the problem and solution of consent on communicative acts rather than on speech acts solely, this new approach to consent found the need to consider communicative actions and egalitarian dialogue for consenting to sexual-affective engagement. Further, Torras-Gómez et al. advanced evidence on the influence of the coercive dominant discourse on young women's pleasure in sexual-affective relationships. After interviewing 13 Spanish women, they found that those who were once influenced by such discourse but later on rejected it felt more pleasure in egalitarian relationships than in power ones, opening up the possibility to find pleasure in falling in love.

Similar to the field of education, psychology has also informed effective interventional programs. Many of those are in the field of gender-based violence. Racionero-Plaza et al. reported the potential social impact of a program consisting of seven interventions addressed to adolescents, and this was grounded in research evidence about the preventive socialization of gender violence. Qualitative and quantitative data from 126 Spanish adolescents revealed the interventions' impact in raising critical consciousness about the influence of the coercive dominant discourse in the adolescents' own life, in better understanding their own sexual-affective relationships, and in transforming their sexual preferences in the direction of rejecting men with violent attitudes and behaviors. In addition, Garzón Segura and Carcedo González designed, implemented, and assessed a gender-based violence prevention program based on evidence from previous successful preventive programs. This quasi-experimental study with 344 primary students in Colombia showed the program succeeded in increasing affective empathy and decreasing gender stereotypes and acceptance of violence.

Beyond sexual-affective violence, psychology has also made important contributions to studies on vulnerable groups and victims of other kinds of violence, for instance, the homeless. Within this line of research, Matulič-Domadzič et al. conducted research into 20 life stories of homeless people in Spain. The authors aimed to identify evidence on solidarity as a key factor in the process of overcoming homelessness and substance abuse situations associated with it. This study showed that a strong solidarity network is essential in overcoming homelessness and impacts participants' well-being and the development of more solidarity attitudes. On the other hand, Wang et al. explored the characteristics and factors influencing violence exposure in real life among 375 Chinese university students who completed three questionnaires. Their analyses showed correlations between deviant behaviors of peers, gender, and single-child status and exposure to violence as well as higher scores in violence exposure in the community than in the family.

Last, two of the studies under this Research Topic showed how and why the methodology used in research in psychology could strengthen its possibilities for achieving social impact. Redondo-Sama et al. focused on social impact assessment methods of psychological research and presented the Communicative Methodology (CM) as a useful methodology for the communicative evaluation of social impact. Their study showed how the CM is increasing the possibility of research in psychology to achieve social impact through a bottom-up approach that includes citizens' voices throughout the entire research. Similarly, Pulido et al. showed the potential of the Social Impact in Social Media (SISM) methodology to evaluate and further promote the visibility of the social impact of psychological research. Through analyzing 10 studies on well-being, their findings confirmed the contributions of the SISM methodology to identify and extract evidence of the social impact of those studies shared on social media, shedding light on how this methodology enables researchers to become aware of whether and how citizens are applying their evidence and establishing improvements.

The findings from these articles establish evidence on how psychological research improves the lives of citizens in a variety of ways, dismantling false information (Pulido et al., 2020; Pulido Rodríguez et al., 2020) about the lack of utility of psychological science and moving toward society's partaking in advancements and benefits of research in psychology.

## Author Contributions

All authors listed have made a substantial, direct and intellectual contribution to the work, and approved it for publication.

## Conflict of Interest

The authors declare that the research was conducted in the absence of any commercial or financial relationships that could be construed as a potential conflict of interest.

## Publisher's Note

All claims expressed in this article are solely those of the authors and do not necessarily represent those of their affiliated organizations, or those of the publisher, the editors and the reviewers. Any product that may be evaluated in this article, or claim that may be made by its manufacturer, is not guaranteed or endorsed by the publisher.

## References

[B1] Aiello E. Donovan C. Duque E. Fabrizio S. Flecha R. Holm, P.. (2020). Effective strategies that enhance the social impact of social sciences and humanities research. Evid. Policy 1:16. 10.1332/174426420X15834126054137

[B2] Besseelaar P. V. D. Flecha R. Radauer A. (2018). Monitoring the Impact of EU Framework Programmes. Expert report. European Union.

[B3] García-Carrión R. Villarejo-Carballido B. Villardón-Gallego L. (2019). Children and adolescents mental health: a systematic review of interaction-based interventions in schools and communities. Front. Psychol. 10:918. 10.3389/fpsyg.2019.00918 31068881 PMC6491840

[B4] Gómez A. Padrós M. Ríos O. Mara L. C. Pukepuke T. (2019). Reaching social impact through communicative methodology. researching with rather than on vulnerable populations: the roma case. Front. Educ. 4:9. 10.3389/feduc.2019.00009

[B5] Pulido Rodríguez C. Villarejo Carballido B. RedondoSama G. Guo M. Ramis M. Flecha R. (2020). False News Around COVID-19 Circulated Less On Sina Weibo Than On Twitter. How To Overcome False Information? Int. Multidiscipl. J. Soc. Sci. 9, 1–22. 10.17583/rimcis.2020.5386

[B6] Pulido C. Redondo-Sama G Sordé-Martí T. Flecha R. (2018). Social impact in social media: A new method to evaluate the social impact of research. PLoS ONE 13:e0203117. 10.1371/journal.pone.0203117 30157262 PMC6114920

[B7] Pulido C. M. Villarejo-Carballido B. Redondo-Sama G. Gómez A. (2020). COVID-19 infodemic: More retweets for science-based information on coronavirus than for false information. Int. Sociol. 9, 107–28. 10.1177/0268580920914755

[B8] Sordé Martí T. Flecha R. Rodríguez J. A. Bosch J. L. C. (2020). Qualitative inquiry: a key element for assessing the social impact of research. Qual. Inquiry. 26, 948–54. 10.1177/1077800420938117

